# Gene Editing and Systems Biology Tools for Pesticide Bioremediation: A Review

**DOI:** 10.3389/fmicb.2019.00087

**Published:** 2019-02-13

**Authors:** Shweta Jaiswal, Dileep Kumar Singh, Pratyoosh Shukla

**Affiliations:** ^1^Enzyme Technology and Protein Bioinformatics Laboratory, Department of Microbiology, Maharshi Dayanand University, Rohtak, India; ^2^Soil Microbial Ecology and Environmental Toxicology Laboratory, Department of Zoology, University of Delhi, New Delhi, India

**Keywords:** systems biology, xenobiotics, bioremediation, metabolomics, pollutant, metabolic network, gene editing

## Abstract

Bioremediation is the degradation potential of microorganisms to dissimilate the complex chemical compounds from the surrounding environment. The genetics and biochemistry of biodegradation processes in datasets opened the way of systems biology. Systemic biology aid the study of interacting parts involved in the system. The significant keys of system biology are biodegradation network, computational biology, and omics approaches. Biodegradation network consists of all the databases and datasets which aid in assisting the degradation and deterioration potential of microorganisms for bioremediation processes. This review deciphers the bio-degradation network, i.e., the databases and datasets (UM-BBD, PAN, PTID, etc.) aiding in assisting the degradation and deterioration potential of microorganisms for bioremediation processes, computational biology and multi omics approaches like metagenomics, genomics, transcriptomics, proteomics, and metabolomics for the efficient functional gene mining and their validation for bioremediation experiments. Besides, the present review also describes the gene editing tools like CRISPR Cas, TALEN, and ZFNs which can possibly make design microbe with functional gene of interest for degradation of particular recalcitrant for improved bioremediation.

## Introduction

Due to ever-increasing world population and their corresponding food commodities also need to be enhanced (Cazalis et al., 2018; Drangert et al., 2018). This is achieved by lowering down the damage to agricultural crops by pests (Schmidt-Jeffris and Nault, 2018). Pests can be weed, herb, insect, rodent, nematode, and microorganisms (bacteria, fungi, and algae) (Bottrell and Schoenly, 2018; Duke, 2018). Pesticides are classified according to their target and show an excellent role in the production of crop yield and lowering down the rate of agricultural losses due to pest (Allmaras et al., 2018). The intensive use of pesticides at unmanageable rate has led to decontaminate the soil, and agricultural runoffs are being biomagnifying water bodies and increase the toxicity level at each trophic level in food web, i.e., DDT (dichlorodiphenyltrichloroethane) (Plattner et al., 2018; Silva-Barni et al., 2018; Thomas et al., 2008). Besides this, pesticidal compounds also have ill effects on health affecting the function of organs and damage the DNA at molecular level leading to neurological diseases and cancer, i.e., azoxystrobin and atrazine (Fatima et al., 2018; Singh N.S. et al., 2018; Vidart d’Egurbide Bagazgoïtia et al., 2018). Pesticides are also reached in foodstuff beginning from the agricultural field to serving tables (Bakirhan et al., 2018; Kumar D. et al., 2018; Sarlio, 2018). Reports are noted encountering the presence of pesticides in fruit juices, milk, seaweeds (food supplement), and other food items (Bedi et al., 2018; Hamedi et al., 2018; Kumar D. et al., 2018; Leprêtre and Merten-Lentz, 2018). Thus, it is mandatory that the use of synthetic pesticides must be lowered down and organic farming should be done (Tal, 2018). The removal and degradation of pesticidal residues are being done with conventional methods of bioremediation (John et al., 2018; Moorman, 2018). Bioremediation points out environmental decontamination of pesticides by microbiological processes whether *in situ* or *ex situ* (Ortiz-Hernández et al., 2018). *In situ* bioremediation (bioventing, biosparging, and bioaugmentation) decontaminates without removal of soil from the site and *ex situ* (landfarming, biopiling, composting, bioreactors, and electrodialysis) treat the unearth soil at the site (Parween et al., 2018). It is an excellent environmental friendly option for degrading the pollutants (Muangchinda et al., 2018). With the advancement of scientific research methodologies, the gene editing and systemic biology tools are being applied in bioremediation of heavy metal, POPs (persistent organic pollutants), petroleum, acid drainage, xenobiotics (Basu et al., 2018; Dai et al., 2018; Gaur et al., 2018; Malla et al., 2018; Boudh and Singh, 2019). Bioremediation is the involvement of chemical components tends to degrade and tangled microbial metabolic web at the contaminated scenario (Nikolopoulou and Kalogerakis, 2018). Systems biology approach provides information about the microbial system (Hall et al., 2018). These microbial systems under different subsets of conditions respond differently (Montiel-Rozas et al., 2018). Microbial interactions within the communities are also observed with systems biology approaches (Kong et al., 2018). Also, this approach is very helpful in understanding the existence of microbes under different environmental conditions of extreme temperature and pressure (Borja, 2018). Omics such as genomics, transcriptomics, metabolomics, and proteomics aid the systems biology studies of microbes for analyzing the genetic level regulation for bioremediation (De Sousa et al., 2018). Advancement in sequencing with high throughput sequencing (HTS) and next-generation sequencing resolve the novel genes involved in biodegradation pathways of various persistent pollutants (Suenaga, 2012; von Netzer et al., 2018).

## Gene Editing Tools

**FIGURE 1:**
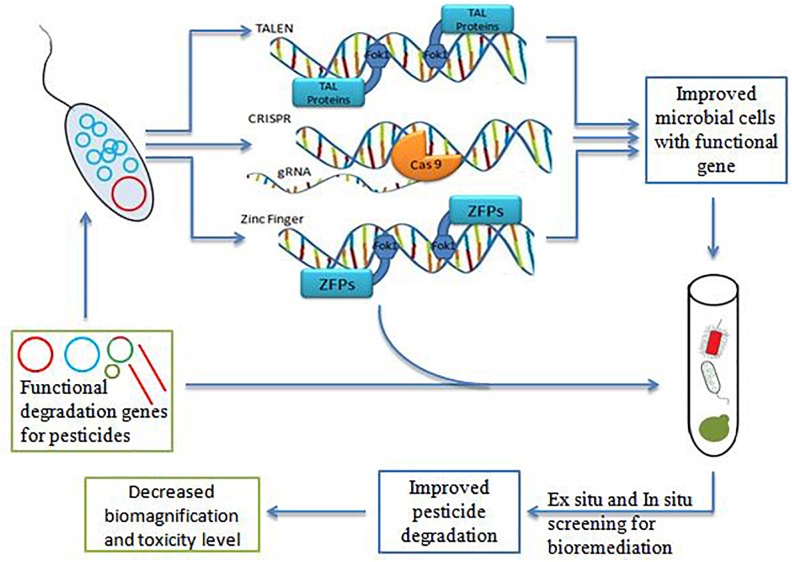
Gene editing tools for bioremediation.

Gene editing is remarkable approach having the ability to manipulate DNA by using engineered nucleases named as molecular scissors. Molecular scissors have an immense application in wide range of research areas related to plant, animals, and microorganism (Butt et al., 2018). The process of editing involves targeting by self-designed guide sequence complementary to sequence of gene of interest assisting break at a site, repaired by homologous recombination, making manipulation (insertion or deletion) of desired sequence fragment (Bier et al., 2018). The genome engineering by gene editing tools led to next level application of microorganisms in various areas like feed, food, agriculture and medical, etc. (Yadav et al., 2018). The gene editing tools have potent capacity to improve the bioremediation processes (Figure 1) such as the elimination of xenobiotics, conversion of toxic compounds to less toxic compounds, and degradation of pesticide to simple components (Basu et al., 2018; Hussain et al., 2018).

The main gene editing tools are CRISPR-Cas, ZFN and TALEN can possibly make the above expectations fulfill (Singh V. et al., 2018; Waryah et al., 2018; Wong, 2018). The collective action of these gene editing tools is to establish double stranded break (DSB) in the target gene sequence, repaired via homology – directed repair (HRD) and error-prone non- homologous end joining (NHEJ) pathway (Arazoe et al., 2018; Yadav et al., 2018). Artificial restriction enzymes are utilized by ZFNs and TALEN which cleave the specific target DNA sequence by Zinc finger DNA binding domain and TAL effector DNA binding domain respectively (Banerjee et al., 2018; Shah et al., 2018). These gene editing tools aim to create better microbe having more complex genes and designing microorganism with maximum quality (Basu et al., 2018; Dangi et al., 2018). It is the root point of altered genetic makeup different from wild types for obtaining the desirable new microbes with functional gene of interest (Dai et al., 2018; Stein et al., 2018). The repercussion thus obtained are off target mutations, lethal mutations and risk of accidental or intentional release of modified organism in environment, etc. make the limitation boundary of using the above gene editing tools (Canver et al., 2018).

### CRISPR-Cas

CRISPR-Cas describe the most effective and productive gene editing (McMahon et al., 2018; Yadav et al., 2018). There are three types, i.e., Types I, II, and III (Zhu Y. et al., 2018) and also numerous subtypes of the CRISPR-Cas systems present (Behler et al., 2018). Each system has specific Cas according to system acting, i.e., model organisms (Cooper et al., 2018). Cas9, a DNA endonuclease is guided by RNA to target foreign DNA for obstruction (Mahas and Mahfouz, 2018). CRISPR is 30–40 bp direct repeat sequence separated by spacer sequence complementing the foreign sequence then after processing and transcription crRNA is formed (Zhang C. et al., 2018). The gRNA (guide RNA) are then obtained by CRISPRs (Listgarten et al., 2018). crRNA and Cas protein together form crRNP (Ribonucleoprotein) which introduce a break in DNA/RNA of the intruder (Majumdar et al., 2017). gRNA makes specific binding to the target DNA site constitute the CRISPR particularity and specificity in function (Shah et al., 2018). The gene of interest can be manipulated (deleted or inserted) from the system with the help of CRISPR/Cas9 by introducing double strand break (DSB) at the target site (Shapiro et al., 2018). The suitable expression system is for the attainment of CRISPR-Cas sgRNA sequence, the codon optimizes variant of Cas9 and ideal promoters for transcription of sgRNA and Cas9 (Rico et al., 2018). The CRISPR technique is obtaining attention by molecular biologist due to the reason, it is highly suited with archeal and bacterial systems (Greene, 2018).

### Transcription Activator-Like Effector Nucleases

TALENs stand for Transcription activator-like effector nucleases. It is an innovative tool for gene modification and editing. TALENs have TAL proteins. These proteins are originally secreted from *Xanthomonas*, a pathogenic bacterial genus. TAL proteins are so effective that they can bind to even very short sequence, i.e., 1–2 nucleotides. Furthermore, the nucleases involved are very efficient in binding due to the presence of 34 amino acids tandem repeats. Gene knock out (non-homologous end joining), and gene knock in (Homology directed repair) of the target gene or gene of interest are now preferred with TALENs. Two protein domains, one for sequence cleavage and second for recognizing and binding the very particular and specific site make the TALENs robust gene editing tool. It is applied to many eukaryotic targets like mammalian cells, frogs, zebrafish, rats, and chickens.

### Zinc Finger Nucleases

ZFNs stand for Zinc Finger Nucleases. It is most commonly used endonuclease. These are artificial restriction enzyme. Zinc Finger Nucleases have ZFPs (Zinc Finger Proteins). ZFPs are basically eukaryotic transcription factors having the ability to act as DNA binding domain. ZFNs also have Folk1 (nucleotide cleavage domain) originated from *Flavobacterium okeanokoites*. Numerous ZFPs (usually four to six) surrounds the cleavage domain depending upon the target site. These ZFPs have 18 bp specificity possibly making the accurate target specific gene editing. ZFPs are 30 amino acids long with alpha-helix in opposition to two antiparallel β-sheets. This gene editing tool is mentioned with gene knock out (non-homologous end joining) and knock in (Homology directed repair) for successful prokaryotic and eukaryotic gene editing.

### Advantages and Disadvantages of Gene Editing Tools

Among the above gene editing tools, CRISPR-Cas is cheap, simple, and easy for researchers to apply in comparison with TALENs and ZFNs (Ju et al., 2018). It has the advantage to evaluate the gene interaction and their genetic and phenotypic relationship along with the gene knock out system replaced with another gene of interest (VanderSluis et al., 2018). The limitation of CRISPR-Cas system is off target mutation leading to lethality, genomic disintegration and hindrance in applicability (Sun et al., 2018). Moreover, unlike CRISPR-Cas is more reliable in terms of specificity in target binding, the TALENs and ZFNs lead to strategies for mutagenesis due to random binding to DNA sequence (Stein et al., 2018).

## Keys of Systems Biology

### Biodegradation Network

The execution of computational tools and bioinformatics resources is an advanced approach toward the pesticide bioremediation (Malla et al., 2018; Vanacek et al., 2018). It takes the online platform of biodegradative databases publicly accessed for retrieving information on biodegradation of xenobiotic (pesticide) by microorganisms and biodegradation pathways of persistent chemicals (Nolte et al., 2018). These databases comprise the University of Minnesota Biocatalysis/Biodegradation Database (UM-BBD), Biodegradation Network-Molecular Biology database (Bionemo), Pesticide Target interaction database (PTID), Microbial Genome Database (MBGD), Biodegradative Oxygenases Database (OxDBase), BioCyc and MetaCyc compatible with both windows as well as Linux operating systems (Arora and Bae, 2014). UMBBD-Pathway Prediction database^1^ displays the data concerning microbial biocatalytic reactions and biodegradation pathways (Erythropel et al., 2018) explored for various types of pesticidal compounds as mentioned in Table 1. The ambition of the UM-BBD is to provide data on microbial enzyme-catalyzed reactions that are important for bioremediation (Ellis et al., 2006). Besides, it also gives the information of intermediate compounds obtained during degradation by microorganisms (Dvořák et al., 2017). With the advancement of synthetic pesticides due to pest resistance, PTID was developed by Gong et al. (2012). This database contains annotation of 1347 pesticides and 13738 pesticide target interactions. By text mining PTID aid to design novel agrochemical products and identification of pesticide targets (Gong et al., 2012). Another database is a Microbial Genome Database (MBGD) an open door for comparative investigation at the genomic level used for evaluating the gene arrangement, ortholog recognition, and collection of paralog data (Bhatt, 2018; Selzer et al., 2018). A broad information resource associated to bioremediation and biodegradation is MetaRouter which allows data mining. It is an established database providing the foundation for bioremediation laboratories, consulting biodegradative routes of different chemical compounds persistent in nature (Korjus, 2014). Synthetic pesticides are called as xenobiotics. OxDBase is biodegradative oxygenase database (Arora et al., 2009). Oxygenase, a class of enzyme, transfers the O_2_ for oxidation of chemical compound (Guengerich and Yoshimoto, 2018). Oxidation is responsible for aromatic ring cleavage breaking down the persistent organic compounds of xeno pesticides (Westcott and Cessna, 2018). OxDBase database also aids the biodegradation network by providing information of oxygenase catalyzed reactions (Shah et al., 2012). Biodegradation and bioconversion of recalcitrant compounds by oxygenases make the bioremediation possible (Sharma et al., 2018). Another database named Bionemo (Biodegradation Network Molecular Biology) have entries of sequences encoding for biodegradation genes (BDGs) and their transcription and regulation (Arora and Bae, 2014). The retrieved data is worthy important for robust biodegradation network (Oladipo et al., 2018). Other components/ databases for robust biodegradation network are mentioned in Table 2. Biodegradation pathways of persistent pesticides, i.e., DDT (dichlorodiphenyltrichloroethane), HCH (hexachlorocyclohexane), and ATZ (atrazine) present under different conditions have been studied (Das et al., 2016). Fang et al. (2014) studied the pesticide biodegradation pathways of isolates from marine and freshwater sediments.

**Table 1:** Classification of the pesticides.

S. no.	Classification of pesticide (examples)	Target (examples)	Reference
1	Herbicide (Benazolin, Bentazone, Imazapyr, Atrazine, Triclopyr, Glyphosate)	Herbs (*Cenchrus macrourus, Eragrostis curvula, Kalanchoe delagoensis*)	Guerra-García et al., 2018
2	Weedicide (Borax, Nitrofan)	Weeds (*Galium spurium, Selaginella kraussiana, Alternanthera philoxeroides, Evolvulus nummularius, Verbesina encelioides, Euphorbia thymifolia*)	Paynter et al., 2015; Kaur et al., 2018
3	Insecticide (DDT, BHC, Chloropyrifos, HCH)	Insects (Grasshopper, Aphid, Beetle, Thrips, Mealybug)	Kumar D. et al., 2018; Qian et al., 2018; van Lenteren et al., 2018
4	Rodenticide (Warfarin, Zinc phosphide)	Rodents (Anas, Platyrhynchos, Aves, Sciurus, Tamias, Rattus, Mus)	Hogue et al., 2018; Mushtaq, 2018
5	Nematicide (Phorate, Fenamiphos, Ethoprop, Dibromochloropropane, Carbamate)	Nematodes (*Meloidogyne incognita, M. javanica*)	Mohamed, 2018; Visagie et al., 2018; Warmerdam et al., 2018
6	Bactericide (Difenoconazole, Mefenoxam, Benzovindiflupyr, Mancozeb, Azoxystrobin, Tebuconazole, Copper sulfate, Pehtahydrate)	Bacteria (*Agrobacterium tumefaciens, Clavibacter, Erwinia amylovora, Xanthomonas campestris, Ralstonia solanacearum, Pseudomonas*)	Milijašević-Marčić, 2018; Scala et al., 2018
7	Fungicide (Monozeb, Methasulfocarb, Prothiocarb, Quinacetol, Sulfuryl fluoride, Trichlamide, Zineb)	Fungi (*Fusarium, Rhizoctonia, Pythium, Phytophthora, Trichoderma, Aspergillus, Penicillium)*	Aladdin et al., 2018; Delaney et al., 2018; Halo et al., 2018
8	Algaecide (Diuron, Copper sulfate, Benzalkonium chloride, Cybutryne, Bethoxazin, Dichlone, Endothal, Fentin)	Algae (*Microcystis, Cyanobacteria, Cephaleuros virescens*)	Bishop et al., 2017; Crafton et al., 2018; Vasconcelos et al., 2018; Wonglom et al., 2018


**Table 2:** Biodegradation databases and their significance.

S. no.	Biodegradation databases	Link	Significance in pesticide bioremediation	Reference
1	University of Minnesota Biocatalysis/Biodegradation Database (UMBBD)	https://www.msi.umn.edu/content/university-minnesota-biocatalysis-and-biodegradation-database	Give information about molecular mechanisms involved in biodegradation pathways and tells about biotransformation rules, enzymes, genes, and reactions involved in microbial degradation of xeno pesticidal compounds	Arora and Bae, 2014; Malla et al., 2018
2	Biodegradation Network- Molecular Biology Database (Bionemo)	http://bionemo.bioinfo.cnio.es	Tells about dynamic regulation of metabolic pathways and transcription factors in degradation pathways	Arora and Bae, 2014; Koch et al., 2018
3	Oxygenase Database (OxDBase)	http://crdd.osdd.net/raghava/oxdbase/	Give information regarding oxygenases, i.e., aromatic ring-hydroxylating dioxygenases (ARHD) and aromatic ring cleavage dioxygenases (ARCD) involved in breaking down pesticidal compounds	Arora and Bae, 2014; Wołejko et al., 2016
4	Pathway/Genome Databases (BioCyc)	https://biocyc.org/	Enable access to information related to biochemistry and genetics of microbial degradation	Arora and Bae, 2014
5	Metabolic Pathway Database (MetaCyc)	https://metacyc.org/	Predict metabolic pathways and reconstruction of catabolic pathways	Millacura et al., 2017; Ali et al., 2018
6	Pesticide Target Interaction Database (PTID)	http://lilab.ecust.edu.cn/ptid	Interaction of pesticides with their target	Ning et al., 2018
7	Microbial Genome Database (MBGD)	http://mbgd.genome.ad.jp	Comparative analysis of microbial genome	Fory et al., 2014; Uchiyama, 2017
8	Metarouter	http://pdg.cnb.uam.es/MetaRouter	Maintain diverse information related to biodegradation	Paliwal et al., 2012; Kumavath and Deverapalli, 2013
9	Pesticide Action Network (PAN)	http://pesticideinfo.org/Index.html	Give informative data on the toxicity of pesticides	Echeverría-Sáenz et al., 2018; Zhu J. et al., 2018
10	The Environmental Contaminant Biotransformation Pathway (EAWAGBBD/PPS)	https://envipath.org/	Give informative from bulk data of multi-omics approaches	Malla et al., 2018


### Computational Tools

**FIGURE 2:**
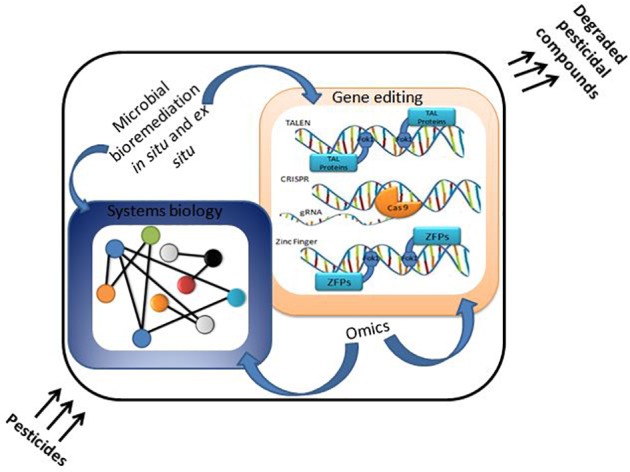
Advancement for pesticide bioremediation through gene editing tools and systems biology.

**FIGURE 3:**
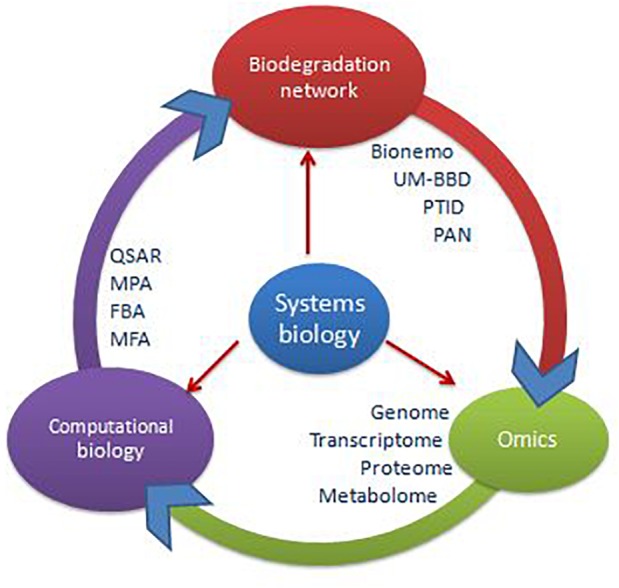
Integrated approaches of systems biology tools in biodegradation network.

Upward elevation of scientific technologies and system biology approaches (Figure 2) represent tools to investigate the interaction of microbe with chemical compounds and their application for bioremediation (Basu et al., 2018). The integrative approach of various computational methods can be applied for the betterment of the bioremediation process to improve soil health (De Sousa et al., 2018). These *in silico* approaches are helpful in the construction of contemporary enzyme based mechanisms for bioremediation (Malla et al., 2018). Computational biology is *in silico* approach for genes and proteins study and dealing with cell system (Purohit et al., 2018). It is feasible to perceive complex metabolic pathways of biodegradation and bioremediation by computational techniques (Liu et al., 2018). *In silico* metabolic engineering of microbes has been done in the various field of microbiology related to agriculture, medical as well as industrial (Babar et al., 2018). There are many *in silico* tools accessible which are used by users for data mining and understanding the metabolic pathways of a cellular metabolic network applied to foster cellular processes, i.e., biodegradation and bioremediation (Ostrem Loss and Yu, 2018; Ravikrishnan et al., 2018; Zhang S. et al., 2018). Flux balance analysis (FBA), metabolic flux analysis (MFA), and metabolic pathway analysis (MPA) are most widely used tools for stoichiometric analysis of metabolic networks (Zhang and Xiu, 2009; Gonzalez-Garcia et al., 2017). Flux can be described as the flow of material with the edges carrying a value (Gomez and Barton, 2018). Knowing flux and organizing it led to alter the biological process dynamics by metabolic engineering (Cuperlovic-Culf, 2018). The consumption of pesticide compounds can also be enhanced, and properties of degrading bacteria can also be manipulated (Sulpice and McKeown, 2015). The reconstruction of quantitative structure-activity relationship (QSAR) and 3DQSAR (Dreher et al., 2018) of chemical atoms created models to assume and predict the interactions of pesticidal compounds with bioremediating and degrading microbes at the molecular level. For instance, QSAR and 3DQSAR is applied to study the toxicity level of xeno-pesticidal compounds at different environmental conditions, i.e., marine ecosystem, terrestrial ecosystem and accumulation or biomagnification of pesticides (DDT) in the food web. 3DQSAR also aids the molecular level study of atomic interactions with different atoms, ligands, and compounds. OptKnock is another interesting computational tool for not only to get gene knock outs but also to get incorporate the genes encoding the novel enzymes for bioremediation as well. A snapshot of integrated approaches of systems biology tools in biodegradation network is depicted in Figure 3. This gene mining from a diversity of microbial strains would be done for integration in a particular and specific microbial GEM (Genome Scale Model) is possible by OptStrain. Moreover, OptReg allows *in silico* regulation and manipulation (positive or negative) of metabolic pathways and enzymes involved in the pathway for functional pesticidal bioremediation efficiency. The above mentioned computational tools help to account for the understanding of the vast amount of genome scale models, their interacting genes and genomic data.

### The Multi Omics Approach

**FIGURE 4:**
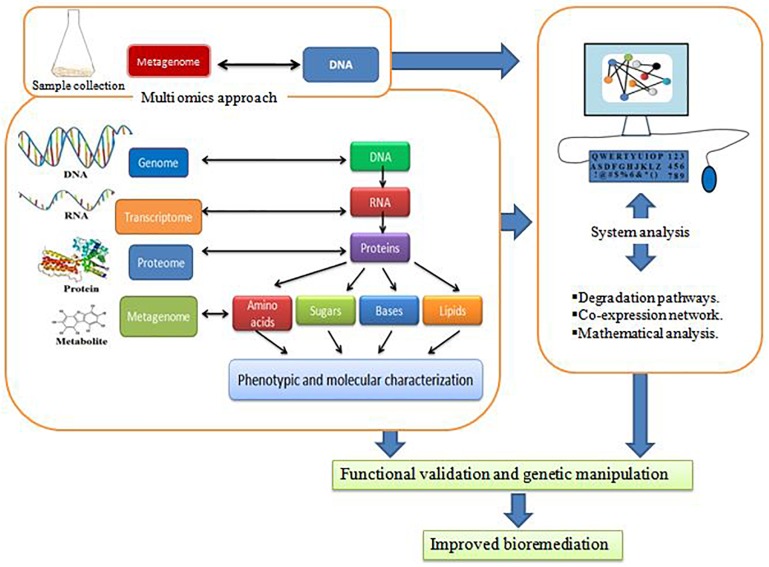
Multi omics approach for improved bioremediation.

With the revolution of computer applications to every possible biological study, it is possible to study the interactions of genes encoding proteins within a cellular model or model organisms via multi omics approach. Thus, it makes possible to study metabolic pathways of biodegrading microorganisms. Genomics deals with the study of DNA for various molecular genetics approaches. These traditional approaches are also applied in the field of bioremediation. Table 3 shows the genomics tools for studying bioremediation of different contaminants in the environment. Environmental scientists consider the metagenomics as the ladder for stepping up in the field of bioremediation (Guerra et al., 2018; Jeffries et al., 2018; Plewniak et al., 2018; Roy et al., 2018). They mentioned the metagenomic appeal for finding the microbial potential degrading heavy metals, oil, petroleum, and other hydrocarbons (Bacosa et al., 2018; Guerra et al., 2018; Logeshwaran et al., 2018; Napp et al., 2018; Ramadass et al., 2018). Metagenomics is the direct analysis of genome (Zolfo et al., 2018). The microbial DNA is extracted directly from the soil sample (Gupta et al., 2018; Jeffries et al., 2018). This repute of metagenomics is the major advantage enabling the DNA analysis of non-culturable microbes present in the sample, i.e., culture independent approach (Perito and Cavalieri, 2018). The DNA thus obtained is sequenced and analyzed for expression (De Sousa et al., 2018). This appeal is now becoming an extension of all life sciences research (Tekle et al., 2018). Metaproteomics is the protein study derived from environmental samples (Biswas and Sarkar, 2018; Bhatt, 2018). Recent reports highlighted metaproteomics approach to observe the bacterial adaptation tactics in various contaminated sites, i.e., heavy metals, oil, xenobiotics, POPs, and other pollutants (Covino et al., 2016; Gianfreda and Rao, 2017; Das and Osborne, 2018). Besides this, the effect of these contaminants on bacterial communities can also be revealed (Mason et al., 2014). Gillan et al. (2015) observed that heavy metal contaminated sites harbouring bacterial community have diversity at the genetic level in terms of release of exopolymers and enzymes. The fast evolution of gene sequencing technology, i.e., HTS has mentioned a vast number of microbes with biodegradation potential. Also, it is adopted with metagenomics to screen particular metagenome of interest, i.e., detection of interacting bacterial species in a community (Gao et al., 2018; Mesuere et al., 2018). Fang et al., 2014 studied biodegradation pathways of persistent pesticides present in ecosystems of marine and freshwater sediments. They did analysis of metagenomic DNA for determining the BDGs involved in degradation of three pesticides namely DDT (dichlorodiphenyltrichloroethane), HCH (hexachlorocyclohexane), and ATZ (atrazine) and created data of 3 giga base pairs. They generated clean data after reducing the garbage data after HTS. Then clean data was analyzed. They get the functional gene annotates of DDT degradation (rrat, cpo, dhc, sds, dcl, ods, dhg, hdl, doa, rdh, hdt, dhc, and ort), HCH degradation (*ccd, dog, dcn, rdg, hdg, cbd, rdt, mog, dhg*, and *dhc*) and atrazine degradation (*atza, atzB, atzC, atzD, thc, apobec, triA, trzA*, and *trzB*). The genome annotation now allowed the identification of functional genes involved in bioremediation and biodegradation. Furthermore, it also enhances the description of existing metabolic pathways for consumption of pesticidal compounds as substrate or metabolites. There are several bacterial strains known for pesticide bioremediation whose whole genome has been sequenced, i.e., *Pseudomonas putida* and *Rhodococcus* sp. Transcriptomics, proteomics and metabolomics study (Table 4) data make us to understand the genotype and phenotype of particular biodegrading microbes. This prediction aids in determining genome scale model (GEM). This model would give the best microorganisms for bioremediation of pesticides and other xenobiotics. These GEMs have created possibilities to utilize the bacterial species with bioremediation potential, i.e., *Pseudomonas putida KT2440* with functional genes (*mpd, opd, vgb, gfp, pnpA, linC, pnpB, linB, linD, and linA*, etc.) (Gong et al., 2016) incorporated for achieving the greater rate of bioremediation at different conditions of pH, temperature, and even at different ecosystems. The above mentioned omics constitutes the multi omics approach (Figure 4) whose action is possible via system analysis. The system analysis would give output to researchers as functional validation and genetic manipulation for improved and efficient bioremediation of contaminants.

**Table 3:** Genomics tools for studying bioremediation of different contaminants.

S. no.	Tool	Purpose	Microorganisms involved	Contaminant	Reference
1	Cloning and sequencing of ribosomal DNA	Identification of ^#^BGD genes in community members of contaminated sites	*Stenotrophomonas maltophilia*	Pesticides, Heavy metals, Acid mine drainage	Raman et al., 2018; Simfukwe and Tindwa, 2018; Shukla et al., 2018
2	Second generation sequencing	Identification of community members having ^#^BGD genes	*Cycloclasticus, Pseudomonas, Halomonas, Pseudoalteromonas, Marinomonas, Bacillus, Dietzia, Colwellia, Acinetobacter, Alcanivorax, Salinisphaera*, and *Shewanella*	Polycyclic aromatic hydrocarbons (PAHs)	Dong et al., 2015; Lozada and Dionisi, 2016
3	Quantitative PCR (polymerase chain reaction), RT-qPCR (real time quantitative PCR)	Quantification of ^#^BGD genes and their expression	*Pseudomonas* and *Rhodococcus*	Diesel	Yergeau et al., 2012; Denaro et al., 2014
4	RFLP (restriction fragment length polymorphism), fingerprinting methods	Bacterial communities involved in biodegradation of persistent compounds	*Thermoanaerobacteraceae, Desulfobulbaceae*	Naphthalene	Marozava et al., 2018
5	FISH (fluorescent *in situ* hybridization)	*In situ* identification of metabolites involved in bioremediation	*Dehalococcoides*	Chlorinated solvents	Matturro et al., 2012
6	SIP (stable isotope probing)	Uptake of labeled compounds as substrate under defined conditions	*Rhodoplanes, Kaistobacter, Pseudomonas, Flavobacterium, Mycobacterium*	Naphthenic acids, phenanthrene, and atrazine	Fenner et al., 2013; Ahad et al., 2018; Lin et al., 2018; Subashchandrabose et al., 2018


*^#^BGD, biodegradation.*

**Table 4:** The multi omics applied for bioremediation study.

S.no.	Omics approach	Center of study	Method	Marker	Application	Microorganism involved	Reference
1	Genomics	Genomic study	DNA sequencing	Gene promoters	Polycyclic aromatic hydrocarbons (PAHs), organophosphate, para-nitrophenol, and phenanthrene compounds of pesticides	*Mycobacterium, Rhodococcus wratislaviensis strain 9*	Birolli et al., 2018; Li J. et al., 2018; Subashchandrabose et al., 2018
2	Metagenomics	Genetic study of sample	Sequencing and pyrosequencing	16S rDNA	Oil, xenobiotics, and heavy metals	*Marinobacterium, Marinobacter, Cycloclasticus, Sphingomonas, Candidatus Solibacter, Flexibacter, Arthrobacter* sp*., Pseudomonas putida, Alcaligenes eutrophus, Dehalospirilum multivorans*	Desai et al., 2010; Dos Santos et al., 2011; Gołêbiewski et al., 2014
3	Metabolomics	Metabolites study of cellular reactions	HPLC, GC–MS	Metabolites	Insecticides, i.e., diazinon, malathion, chlorpyrifos, permethrin, cyfluthrin, cypermethrin, deltamethrin, and pyrethroids	*Streptomyces aureus strain HP-S-01, Bacillus megaterium JCm2, Sphingobium* sp. *JQL4-5, Aspergillus niger, Aspergillus terricola*, and *Candida pelliculosa strain ZS-02*	Cycoń and Piotrowska-Seget, 2016; Radford et al., 2018
4	Proteomics	Study of proteins and their application	X-ray crystallography	Protein, peptides, and oligopeptides	Organophosphorus insecticides	*Aspergillus, Pseudomonas, Chlorella*, and *Arthrobacter*	Kumar S. et al., 2018; Li X. et al., 2018
5	Transcriptomics	Study of transcripts and their function	RNA sequencing, Q- and RT-PCR	miRNA, siRNA, and RNAi transcripts	Organophosphates, pyrethroids, and carbamates	*Pseudomonas putida KT2440, Sphingobium* sp. *strain 1017-1*	Gong et al., 2018; Yan et al., 2018


## Conclusion and Future Perspectives

Dissimal and removal of persistent pesticides by gene editing tools and systems biology have come forth as the outstanding option. Many bioremediation approaches are present to solve the difficulties in the field of bioremediating recalcitrant pollutants from the environment. The environment is constantly being harmed by the continuous use of synthetic pesticides (xeno-pesticidal compounds). These synthetic pesticides are organic and inorganic compounds whose remediation processes vary from one another thus it is obvious that individual or single bioremediation pathway is not enough. Therefore, empathizing on metabolic pathways for gene editing and application of systems biology is very important. This will undertake the existing metabolic pathways towards the increased and efficient microbial remediation of pesticides. Acceptable improvements have been witnessed for bioremediation of pesticides by applying gene tools. Furthermore, genomics, metagenomics, metabolomics, transcriptomics, proteomics, and biodegradation network pave the path of pesticide bioremediation. TALEN, ZFNs, and CRISPR Cas9 are auspicious gene editing tools to get the function specific microorganisms with particular genes and enzymes responsible for pesticide bioremediation. The multi omics approach also contributed to the logical identification of microbial host having degradation strength.

## Author Contributions

All authors listed have made a substantial, direct and intellectual contribution to the work, and approved it for publication.

## Conflict of Interest Statement

The authors declare that the research was conducted in the absence of any commercial or financial relationships that could be construed as a potential conflict of interest.

## Footnotes

1 https://www.msi.umn.edu/content/university-minnesota-biocatalysis-and-biodegradation-database
